# Supplementary material for: Historical biogeography of the leopard (Panthera pardus) and its extinct Eurasian populations
Source: BMC Evol Biol. 2018 Oct 23;18:156. doi: 10.1186/s12862-018-1268-0 (PMC6198532; doi:10.1186/s12862-018-1268-0)
Supplement: Supplementary file 1 — Figure S1. Maximum likelihood phylogeny, with different Panthera species as outgroup. Figure S2: Minimum-spanning network of short (456 bp) mtDNA sequences (ND5 gene), including previously published data (based on 267 sequences in total). Number of substitutions are indicated as tick-marks on the branches connecting the haplotypes. Colours indicate the subspecies [19, following 25]. Table S1: Summarised sequence statistics for samples included in our study. Table S2: Fossil constraints and calibration priors used in the time-calibrated BEAST analysis performed for Felidae alignment. The resulting root age was then applied as calibration for the leopards-only phylogeny. Table S3: Radiocarbon dating (14C) information. (PDF 1852 kb) [file 12862_2018_1268_MOESM1_ESM.pdf]

Historical biogeography of the leopard (*Panthera pardus*) and its extinct Eurasian populations

Johanna L.A. Paijmans, Axel Barlow, Kirstin Henneberger, Daniel W.G. Förster, Matthias Meyer, Birgit Nickel, Gennady F. Baryshnikov, Ulrich Joger, Wilfried Rosendahl, Doris Nagel, Rasmus Worsøe Havmøller, Michael Hofreiter

Additional File 1

Figure S1: Maximum likelihood phylogeny, with different *Panthera* species as outgroup.

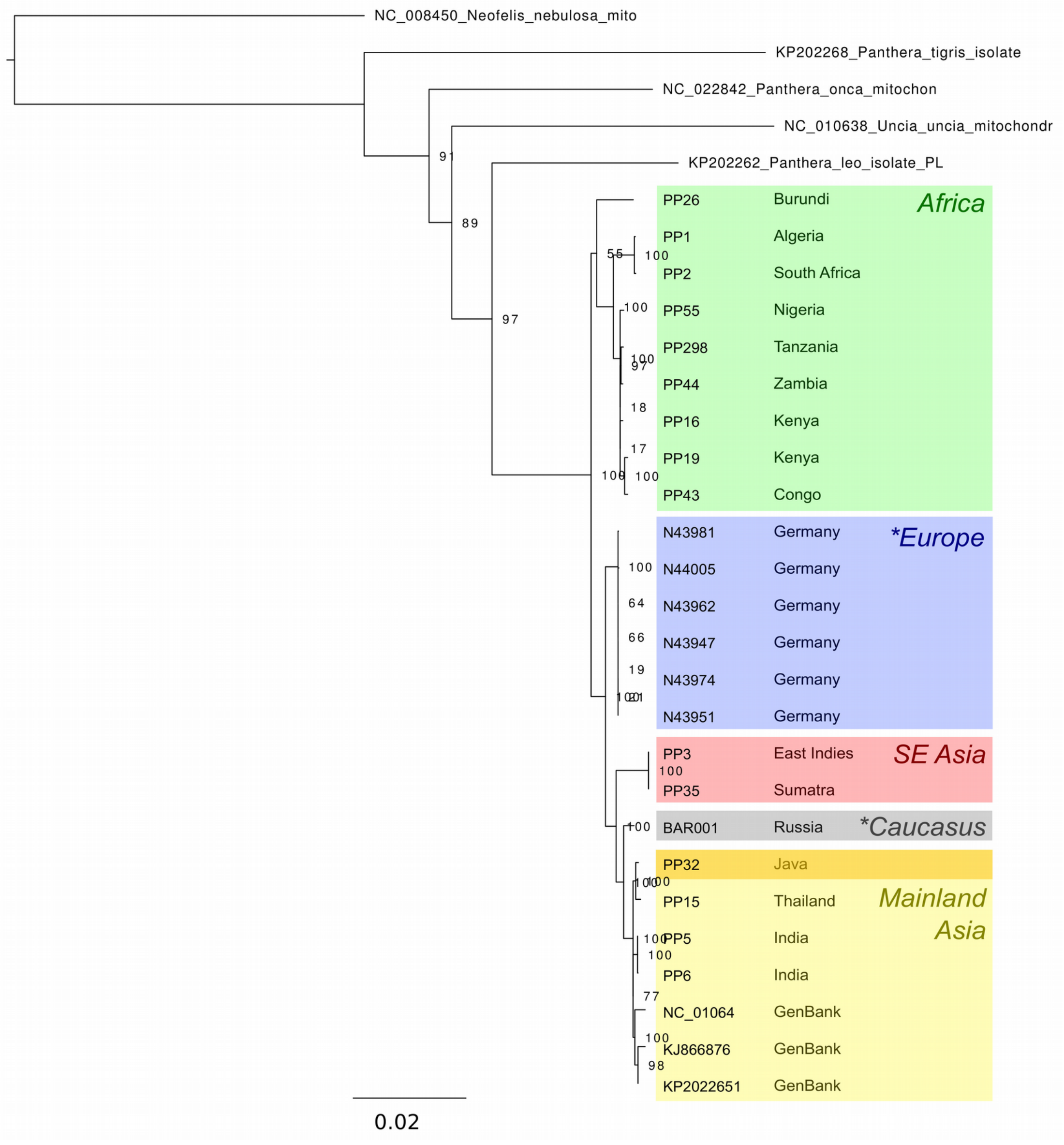

Figure S2: Network of short (456 bp) fragment mtDNA (ND5 gene), including previously published data (based on 267 sequences in total). Number of substitutions are indicated as tick-marks on the branches connecting the haplotypes. Colours indicate the subspecies (following Uphyrkina et al., 2001).

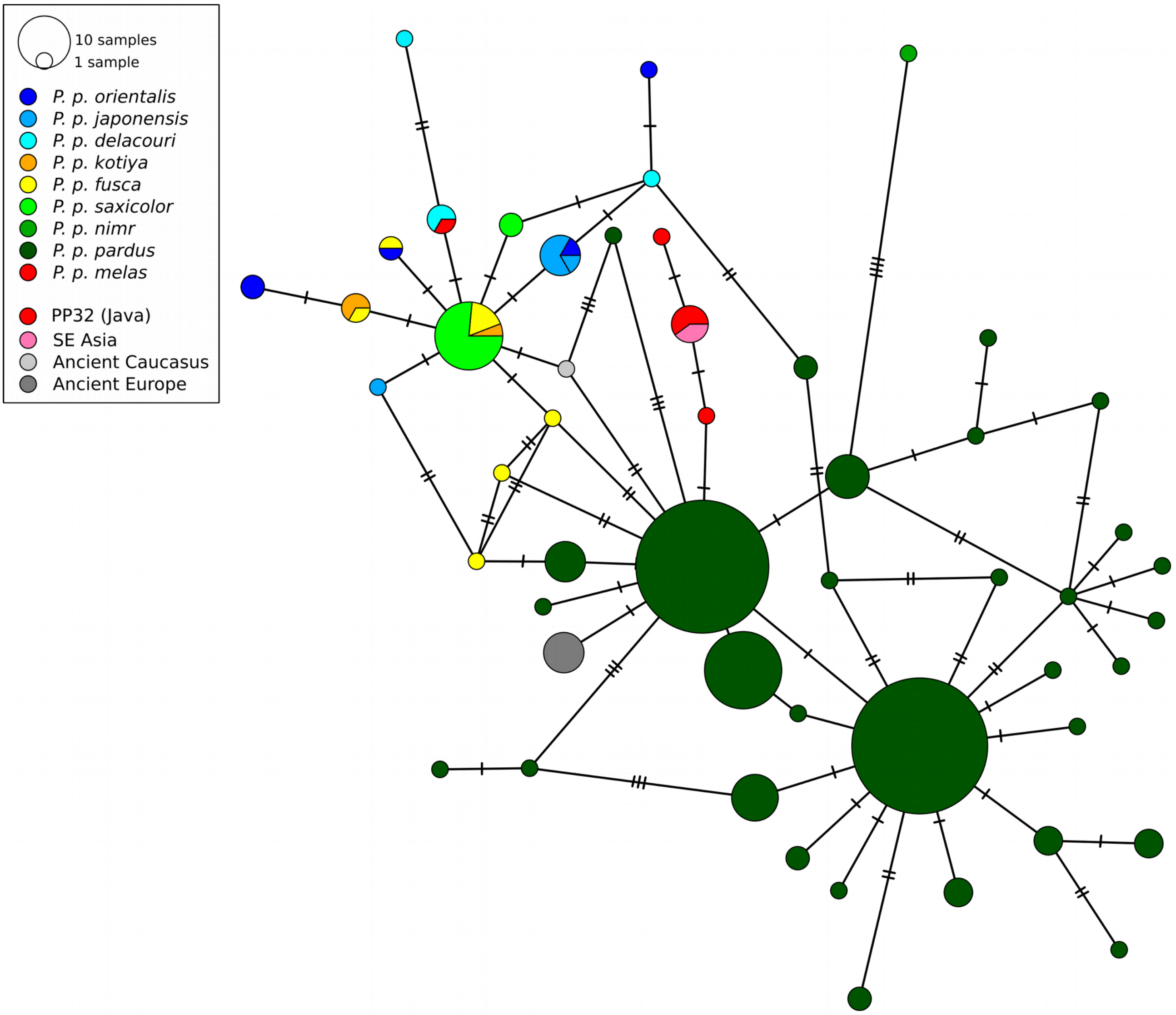

Table S1: Summarised sequence statistics for samples included in our study.

Sample ID	Nr_reads merged (seqprep)	Nr_reads aligning	Nr_reads aligning after rmdup	mean_coverage_depth	percentage_over_15x	%_mito_>2x
**Ancient capture:**						
BAR001-cap2	5,282,537	2,238,082	93,758	314.52	99.7	99.89%
N43951-cap2	3,557,529	997,929	22,649	52.2	91.1	98.76%
N43962-cap2	3,683,484	1,069,833	21,873	49.22	89.7	98.27%
N43974-cap2	4,782,338	1,208,060	22,305	53.02	92.7	98.73%
N43981-cap2	4,728,170	944,769	7,962	18.45	50.1	94.85%
N44005-cap2	3,725,267	1,512,124	11,084	26.03	77.9	98.06%
N43947-cap2	3,563,518	484,795	8,251	19.73	57	96.06%
**Historical shotgun:**						
leo-PP19	23,636,029	34,521	33,163	146.84	95.3	98.45%
leo-PP32	27,155,197	8,137	7,949	34.07	93.1	99.79%
leo-PP44	12,748,947	5,172	5,062	23.31	78.6	94.93%
leo-PP5	24,632,610	11,663	11,374	47.55	97.1	99.45%
**Historical capture:**						
PP1-cap2	8,428,050	1,780,262	974,381	4226.33	99.4	99.68%
PP15-cap	7,399,879	3,387,087	13,045	64.24	99.8	99.96%
PP16-cap2	6,845,173	1,780,262	700,530	3307.66	99.4	99.65%
PP2-cap2	7,962,286	4,752,083	666,768	2799	99.3	99.50%
PP26-cap	6,606,406	2,308,481	914,522	4553.08	99.8	99.79%
PP298-cap2	4,920,382	1,868,918	334,589	1967.92	98.1	99.52%
PP3-cap	8,378,965	3,888,526	128,455	548.2	99	99.68%
PP35-cap2	7,095,539	3,123,992	771,634	4013.24	99.5	99.69%
PP43-cap2	7,152,437	2,382,558	599,103	2966.61	98.9	99.21%
PP55-cap	6,493,490	2,277,276	54,877	278.44	96.7	99.23%
PP6-cap	7,840,609	5,008,104	701,180	3132.21	100	100.00%
**Negative controls**						
liBLK-14-9-15-2-cap2	2	0	0	0	0	0.00%
eBLK-08-09-2015-3-cap3	840,748	59,276	28	0.07	0	1.00%
eBLK-8-9-15-2-cap2	500,019	4,647	5	0.01	0	0.00%
liBLK-15-9-15-cap2	443,137	0	0	0	0	0.00%
PP-eBLK-28-10-15-2	77	11	11	0.04	0	0.00%
PP-liBLK-17-11-15-1	74	18	4	0.02	0	0.00%
PP-liBLK-17-11-15-2	70	0	0	0	0	0.00%
PP-liBLK-22-9-15-l	200,771	0	0	0	0	0.00%
PP-liBLK-23-09-15-l	215,492	0	0	0	0	0.00%

Table S2: Fossil constraints and calibration priors used in the time-calibrated BEAST analysis performed for Felidae alignment. The resulting root age was then applied as calibration for the leopards-only phylogeny.

	Uniform prior			
**Fossil Calibration**	**Min**	**Max**	**Estimated mean age**	**Reference**
Caracal fossil: 3.8 Ma	3.8 Ma	10 Ma	7.2	van Valkenburg et al. 1990
Lynx fossil: 5.3 Ma	5.3 Ma	10 Ma	6.2	Eizirik et al. 2010, McKenna & Bell 1997
Panthera fossil: 3.8 Ma	3.8 Ma	10 Ma	7.8	van Valkenburg et al. 1990, Turner & Anton 2005
Acinonyx fossil: 3.8 Ma	3.8 Ma	10 Ma	5.1	van Valkenburg et al. 1990
P. onca fossil: 1.5 Ma	1.5 Ma	10 Ma	4.3	Turner & Anton 2005
P. tigris fossil: 1.5 Ma	1.5 Ma	10 Ma	4.3	Turner & Anton 2005
P. uncia fossil: 1.4 Ma	1.4 Ma	10 Ma	3.8	Turner & Anton 2005

Table S3: Radiocarbon dating (^{14}C) information.

Leopard specimen	Labcode MAMS	Identifier	^{14}C Age [yr BP]	±	δ13C AMS [‰]	Cal 1-sigma	Cal 2 Sigma	C:N	C [%]	Collagen [%]	Material type
N43962	31739	GMP 196	44710	630	-3	cal BC 46840-45275	cal BC47597-44612	3.3	22.4	1.1	bone
N43981	31740	GMP 197	37880	300	-12.6	cal BC 40420-39995	cal BC40641-39769	2.9	22	1.9	bone
N44005	31741	GMP 198	40470	410	-24.9	cal BC 42480-41665	cal BC42856-41295	-	-	3.1	bone
